# Supplementary material for: Chronic Alcohol Use Induces Molecular Genetic Changes in the Dorsomedial Thalamus of People with Alcohol-Related Disorders
Source: Brain Sci. 2021 Mar 29;11(4):435. doi: 10.3390/brainsci11040435 (PMC8066746; doi:10.3390/brainsci11040435)

Supplementary figures:

Andreas-Christian Hade, Mari-Anne Philips*, Ene Reimann, Toomas Jagomäe, Kattri-Liis Eskla, Tanel Traks, Ele Prans, Sulev Kõks, Eero Vasar and Marika Väli

Chronic alcohol use induces molecular genetic changes in the dorsomedial thalamus of people with alcohol-related disorders

Figure S1: Immunochemistry controls (A-D) images display specificity of secondary antibody and (E - H) distribution of GRIN1 in AUD subjects´s mediodorsal thalamic nucleus (MDMC). (B, C, D) auto-fluorescence and or unspecific binding of secondary antibodies are detectable in blood vessels and in presumptive lipofuscin granules (also visible in F - H). (F, H) specific staining of GRIN1 is observable in the AUD brain MDMC presence of primary antibody revealing intensely stained puncta.

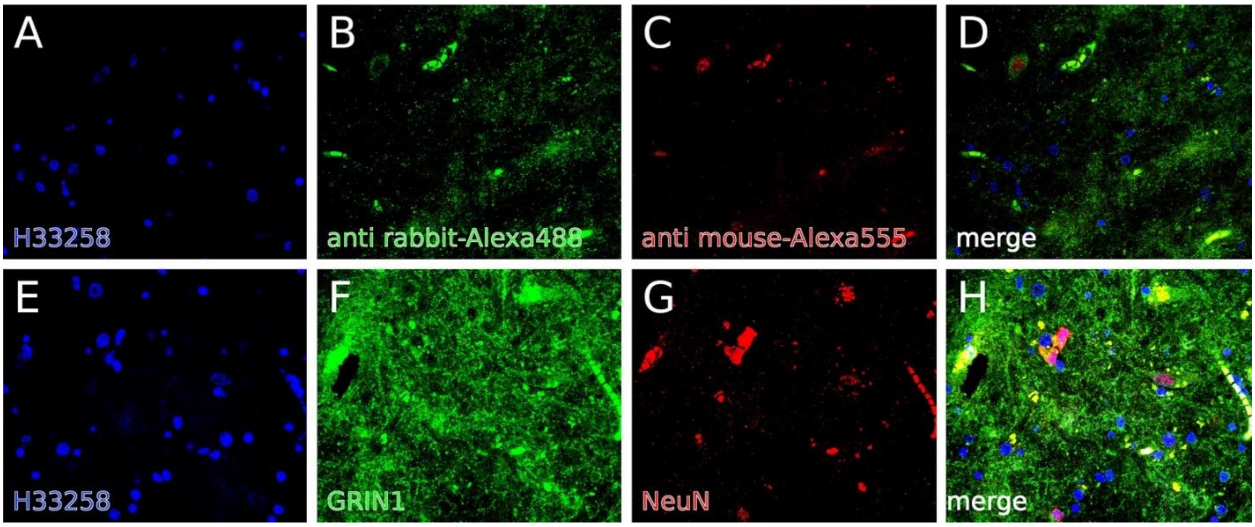

Supplementary figure S2: “TCA Cycle (aka Krebs or citric acid cycle)” (Wikipathways, FDR p = 0,001). Red background of the genes indicates significant upregulation of the gene in the dorsomedial thalamus of the AUD subjects, striped background of the genes indicates genes that have been filtered out.

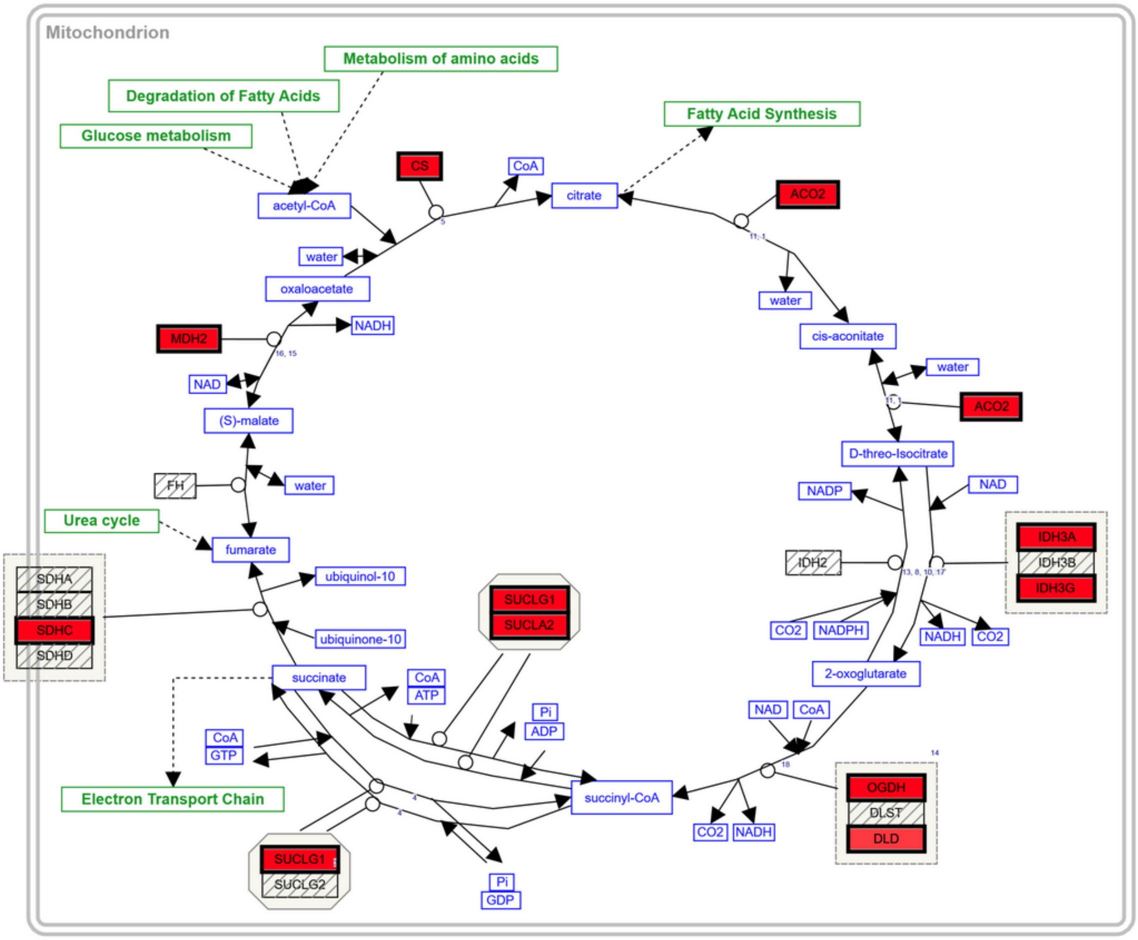

Supplementary figure S3: "Fatty Acid Biosynthesis" (Wikipathways, FDR p < 0,001). Red background of the genes indicates significant upregulation of the gene in the dorsomedial thalamus of the AUD subjects, striped background indicates genes that have been filtered out.

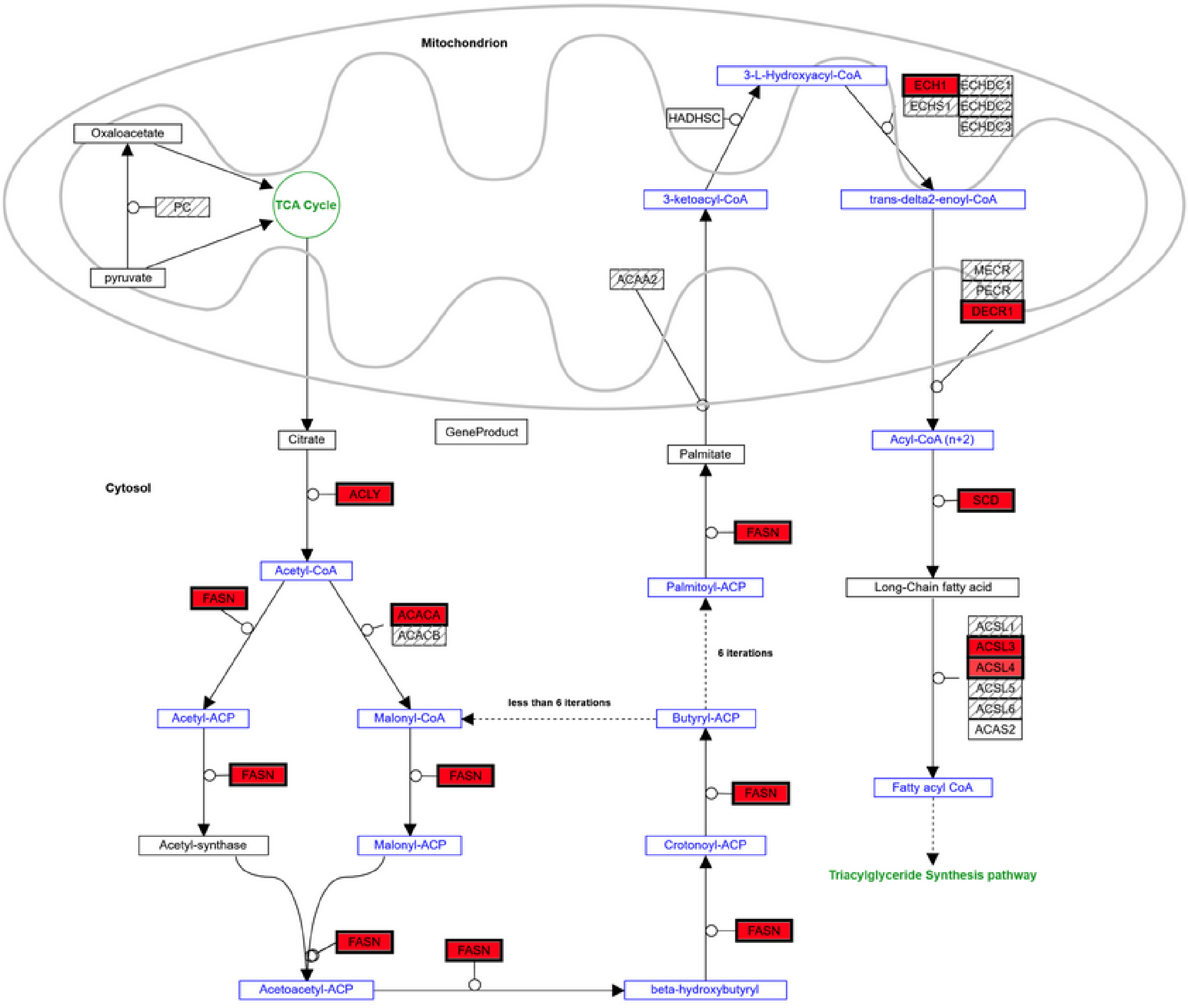

Supplementary figure S4: “GABA receptor Signaling” (Wikipathways, FDR p = 0,001). Red background of the genes indicates significant upregulation of the gene in the dorsomedial thalamus of the AUD subjects, striped background indicates genes that have been filtered out during analysis.

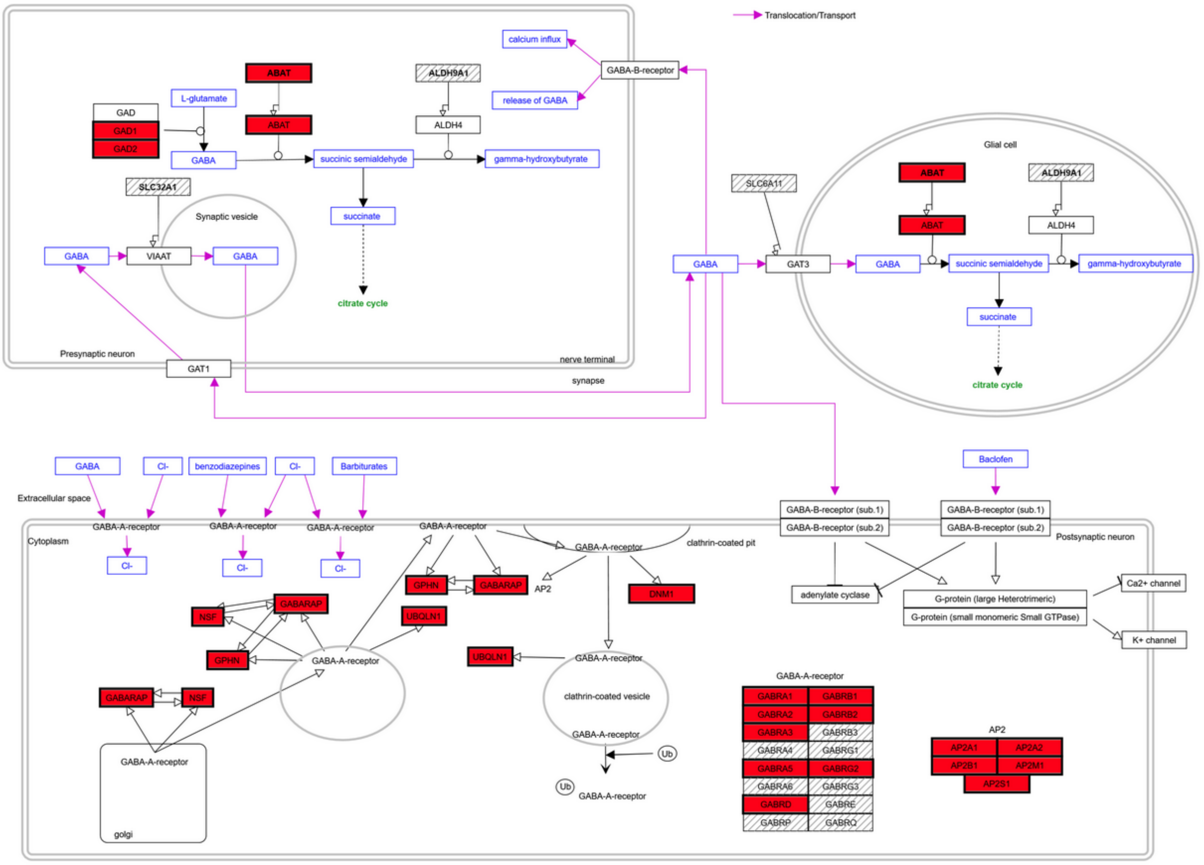

Supplementary figure S5: “Synaptic Vesicle Pathway” (Wikipathways, FDR p < 0,001). Red background of the genes indicates significant upregulation and green back-ground downregulation of the gene in the dorsomedial thalamus of the AUD subjects, striped background indicates genes that have been filtered out during analysis.

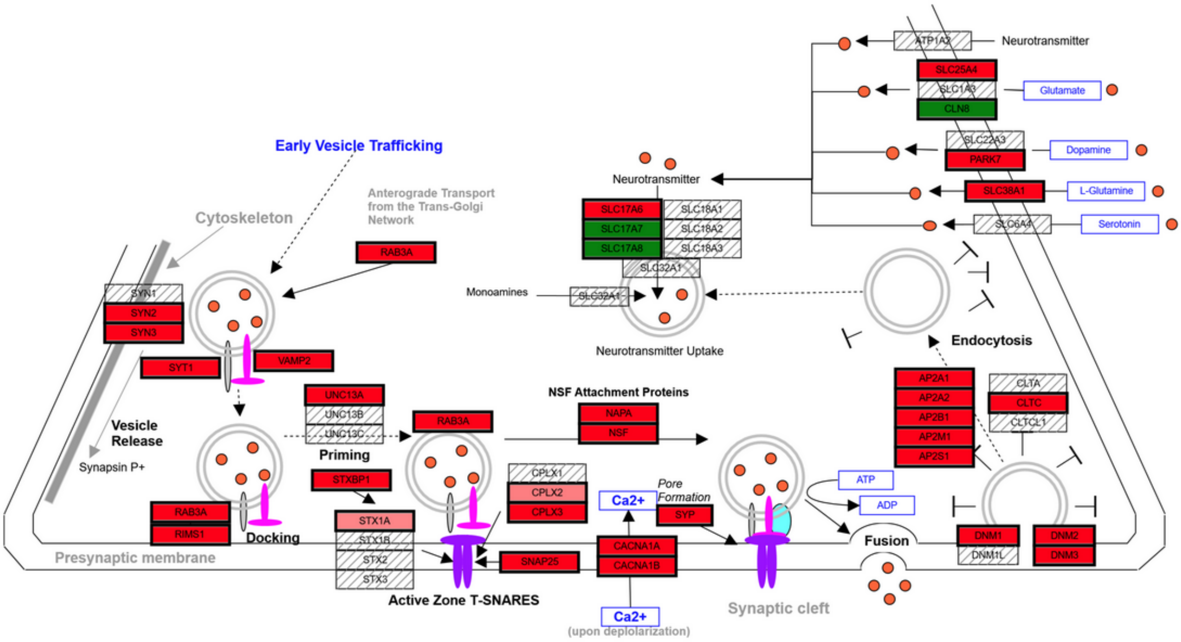

Supplementary figure S6: "Disruption of postsynaptic signalling by CNV (copy number variations)" (Wikipathways, FDR p= 0,0001). Red background of the genes indicates significant upregulation and green background significant downregulation of the gene in the dorsomedial thalamus of the AUD subjects, striped background indicates genes that have been filtered out during analysis.

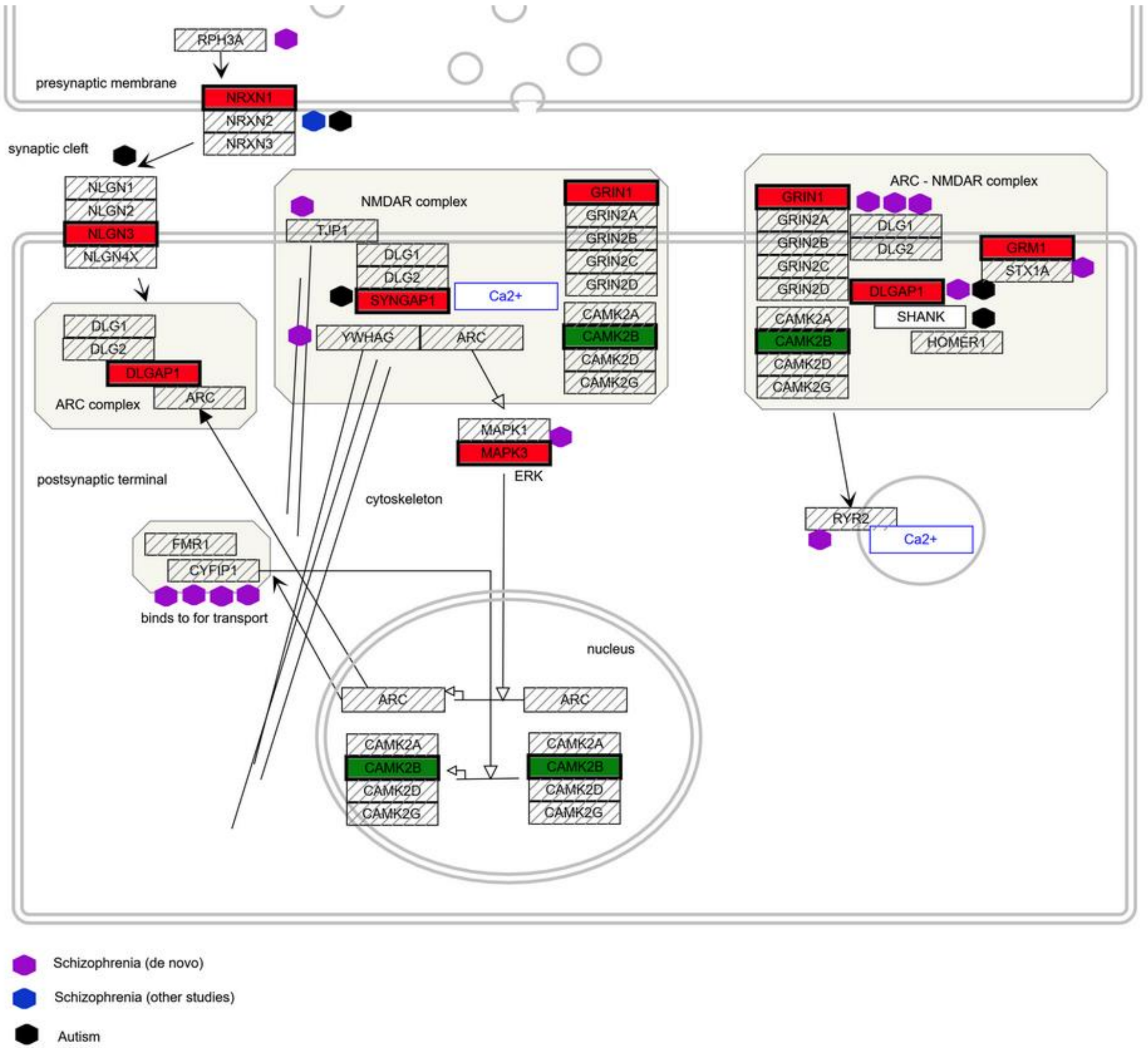

Supplementary figure S7: "G Protein Signaling Pathways" (Wikipathways, FDR p= 0,0001). Red background of the genes indicates significant upregulation and green background significant downregulation of the gene in the dorsomedial thalamus of the AUD subjects, striped background indicates genes that have been filtered out during analysis.

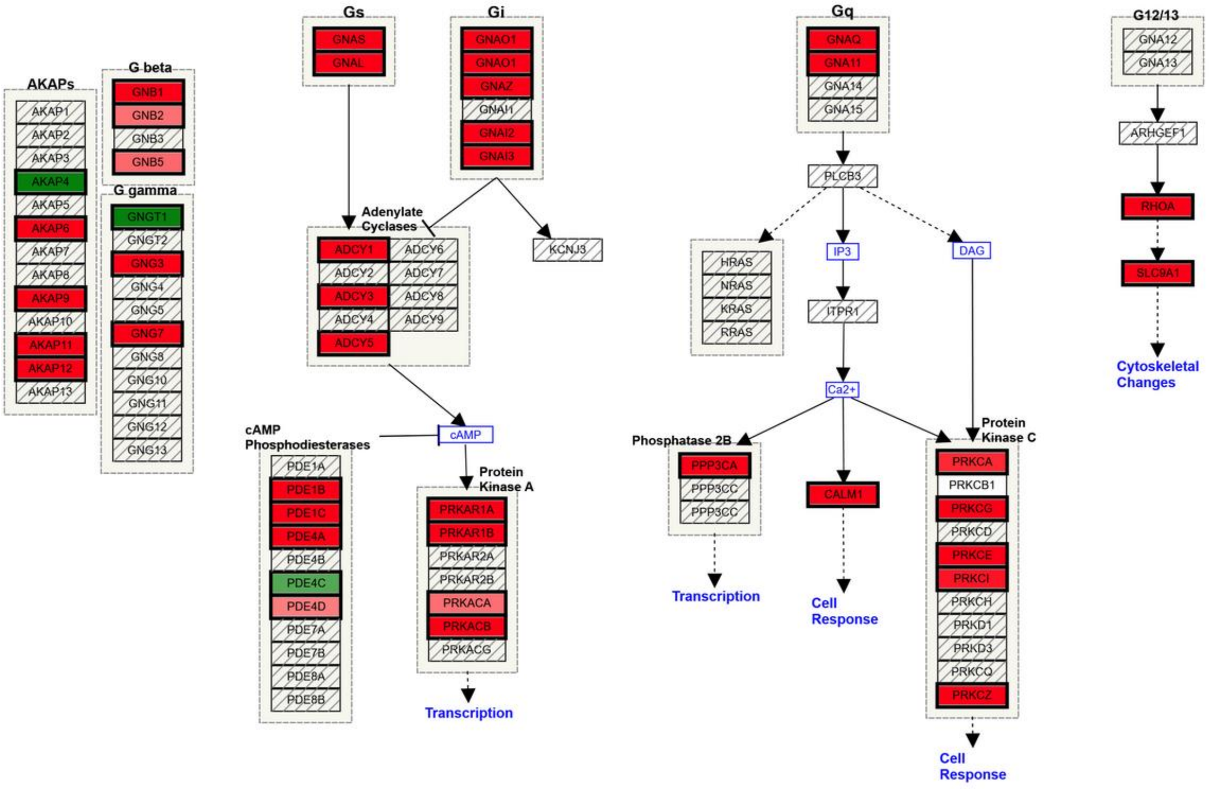

Supplementary figure S8: „Chemokine Signalling Pathway“ (Wikipathways, FDR p= 0,01). Red background of the genes indicates significant upregulation and green background significant downregulation of the gene in the dorsomedial thalamus of the AUD subjects, striped background indicates genes that have been filtered out during analysis. The pathway is depicted partially

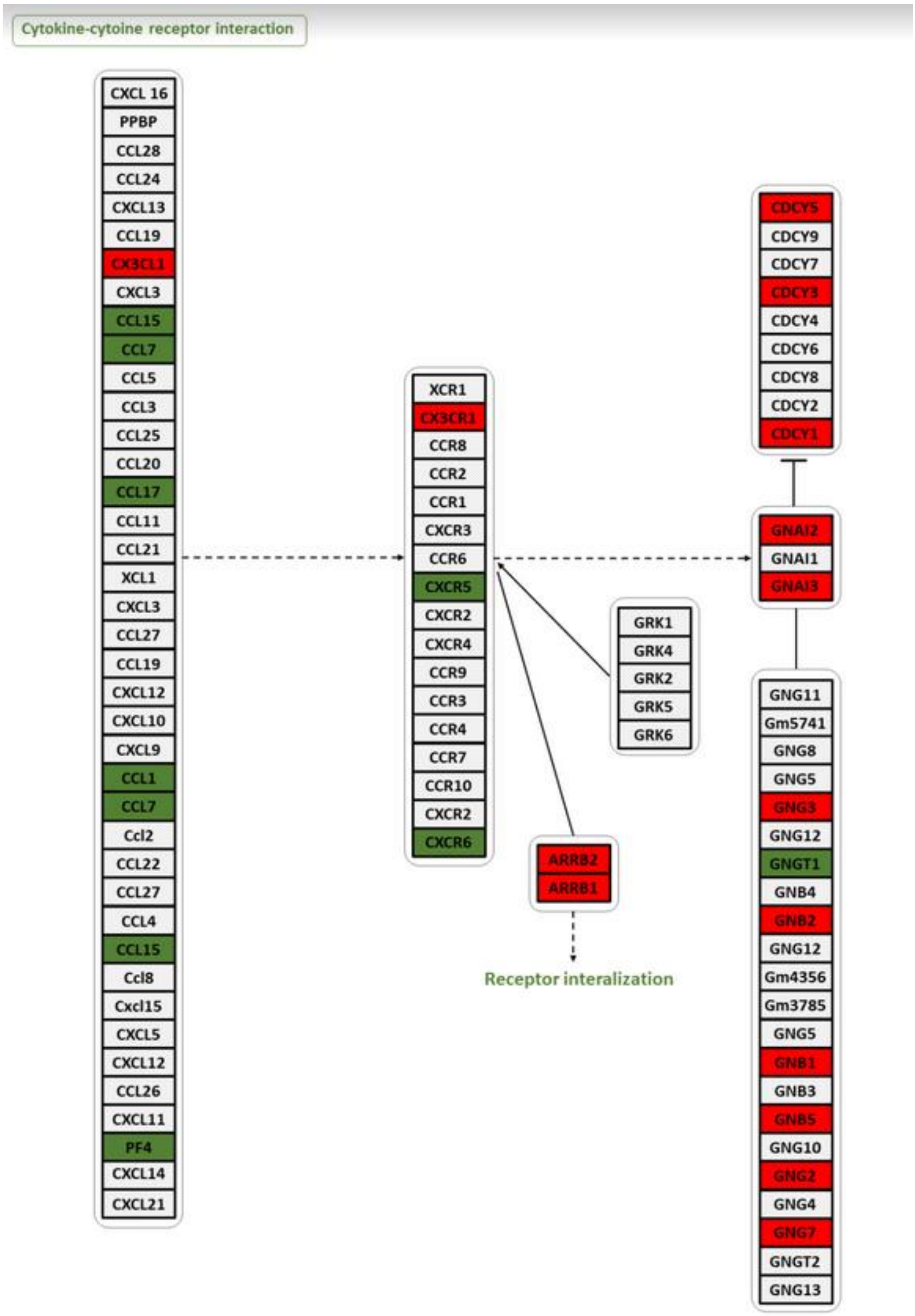

Supplementary figure S9: "Cannabinoid receptor signaling" (Wikipathways, FDR p < 0,05). Red background of the genes indicates significant upregulation and green background significant downregulation of the gene in the dorsomedial thalamus of the AUD subjects, striped background indicates genes that have been filtered out during analysis.

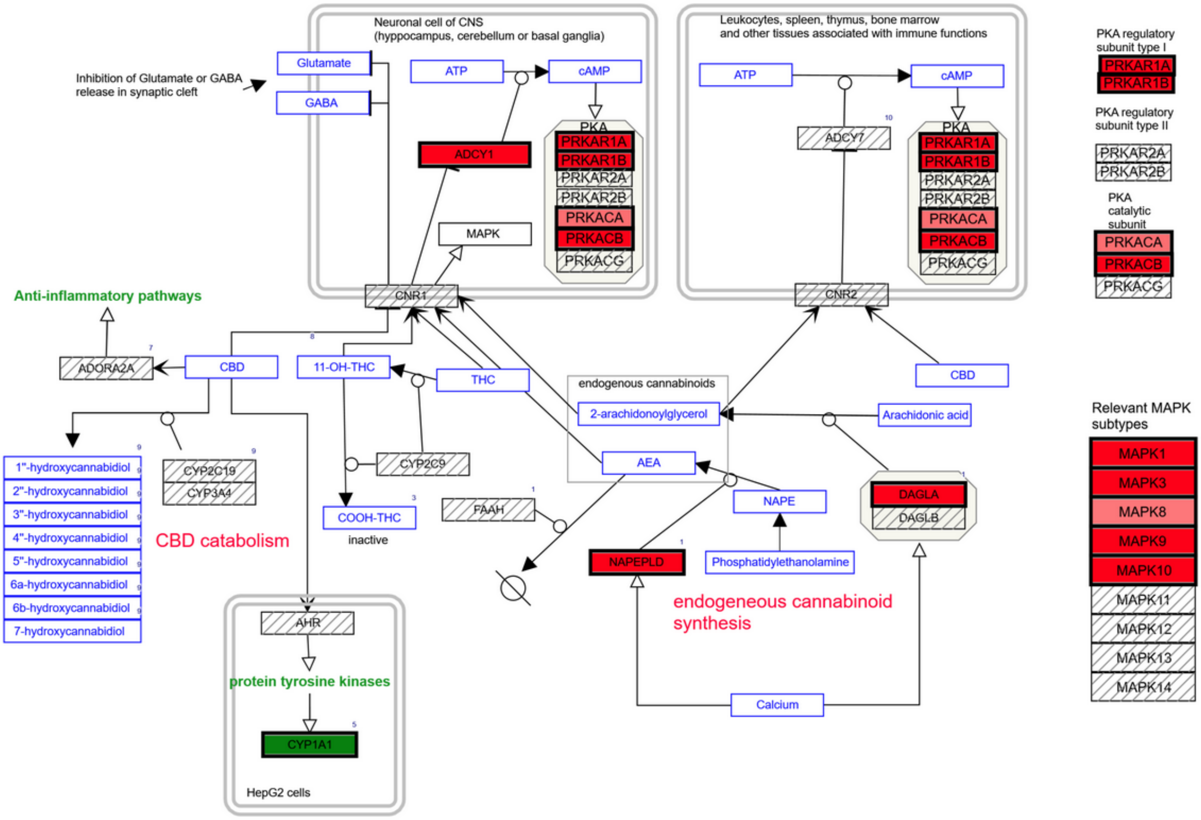

Supplement: Supplementary file 1 [file brainsci-11-00435-s001.zip › supples-brainsci-1145509/Supplementary figures S1-S9.pdf]
